# Magnetism in curved VSe_2_ monolayers†

**DOI:** 10.1039/d3ra01319g

**Published:** 2023-03-14

**Authors:** Kexin Mi, Yufeng Guo

**Affiliations:** a State Key Laboratory of Mechanics and Control of Mechanical Structures, MOE Key Laboratory for Intelligent Nano Materials and Devices, College of Aerospace Engineering, Nanjing University of Aeronautics and Astronautics Nanjing 210016 China yfguo@nuaa.edu.cn

## Abstract

Our extensive first-principles calculations on magnetic VSe_2_ monolayers reveal the curvature-induced periodic fluctuation in the magnetic moments of V atoms and the occurrence of charge density waves for curved VSe_2_ monolayers. The bending energies of curved 2H-VSe_2_ monolayers increase with increasing curvature but that of curved 1T-VSe_2_ monolayers with curvature is not monotonic. The significant periodic magnetic orders in curved VSe_2_ monolayers can be attributed to the curvature-induced modification of V–Se bond structure and periodic length variations in V–Se bonds. A phenomenological model is established to describe the relation of the total magnetic moment in one period of a curved VSe_2_ monolayer with its curvature radius and the number of hexagonal rings that forms one period. These results unveil the effect of bending deformation on magnetic van der Waals monolayers and provide a possible way to develop functional magnetic devices by mechanical design.

## Abstract 2

The curvature-induced periodic fluctuation in the magnetic moments and bond length deviations of V atoms and the occurrence of charge density waves for curved VSe_2_ monolayers.

## Graphical abstract

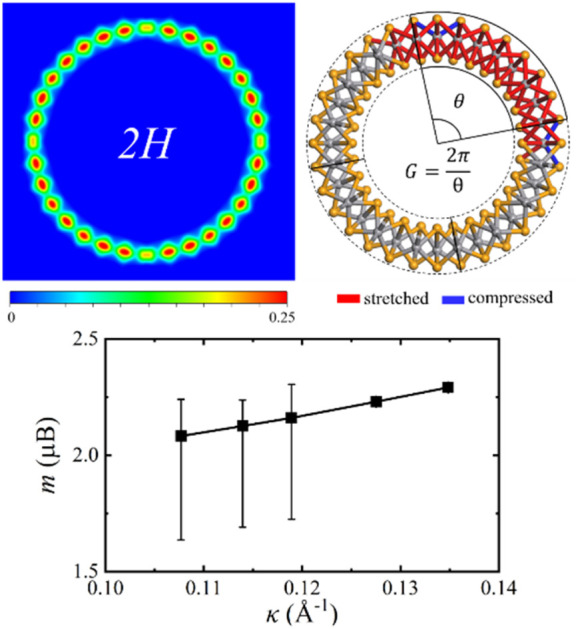

## 1. Introduction

Magnetism in low-dimensional materials has attracted a lot of scientific attention and interest because of the potential applications in information storage technology, spintronics, and valleytronics. Recently, intrinsic magnetism or magnetic order has been observed in two-dimensional (2D) atom layers of FePS_3_,^1,2^ CrI_3_,^3^ Cr_2_Ge_2_Te_6_,^4^ VSe_2_,^5^ and MnSe_2_ (ref. 6 and 7) that were experimentally obtained from chemical vapor deposition, molecular beam epitaxy growth, and mechanical adhesion and exfoliation. Besides experimental methods, a theoretical work^8^ based on the high-throughput computation predicts 56 possible magnetic 2D materials that are held by weak van der Waals (vdW) interactions and easily exfoliated from their bulk states. Magnetic 2D materials extend the fundamental knowledge of magnetism at the nanoscale and provide an ideal platform to design novel magnetic devices and tune the electron and spin behaviors in a few atom layers.

The capability of 2D materials to resist in-plane or out-of-plane deformation is relatively low due to one or a few atom-layer thicknesses. Lattice mismatch-induced strain, wrinkles and ripples, and moiré patterns are commonly observed for substrate-supported 2D materials. External perturbation and structural deformation usually impose a significant impact on the magnetic properties of 2D materials. For example, applying biaxial tensile strain on monolayer VSe_2_ could enhance the magnetic moment of V atoms and the total ferromagnetism. In the experiment, monolayer VSe_2_ has been successfully synthesized and was reported to possess strong room temperature in-plane and out-of-plane ferromagnetism,^5^ but other followed experimental and theoretical works revealed that substrate-induced structural distortion and the presence of charge density wave (CDW) suppress the ferromagnetism of VSe_2_ monolayer and whether the intrinsic ferromagnetism exists in VSe_2_ monolayer is still in debate.^9–16^ On the other hand, the structural deformation on 2D materials not only changes the intrinsic mechanical and physical behaviors of 2D materials but also endows them with new phenomena and properties.^17,18^ The appearance of strain gradient in low-dimensional materials caused by nonuniform mechanical strain or inhomogeneous deformation can have a comparably strong influence on charge polarization and electronic behaviors,^19–24^ spin–orbit interactions,^25,26^ and topological magnetism,^26^ which gives rise to notable flexoelectric and flexomagnetic effects.^24,27–31^ As the low flexural stiffness of thin atomic layers, magnetic 2D materials can be easily bended or deformed under mechanical loading or substrate interaction. The magnetic 2D materials obtained by experimental methods or predicted by theoretical calculations usually possess different magnetism and bond structure. However, the effect of bending deformation on the atom arrangement and bond structure of magnetic 2D materials remains unclear, and it is still necessary to further study the curvature-induced change in the magnetism and electronic structure of magnetic 2D materials.

In this study, the influence of homogenous bending deformation on the magnetism and bond structure of magnetic VSe_2_, MnSe_2_ and CrI_3_ monolayers has been investigated by first-principles calculations. Our results show that the magnetic moments of V atoms of curved VSe_2_ monolayers exhibit periodic fluctuation along the circumference direction and the periodic CDWs occur in curved VSe_2_ monolayers. The bending energies of 2H-VSe_2_ monolayers increase with increasing the curvature but that of 1T-VSe_2_ monolayers with curvature is not monotonic. The remarkable periodic magnetic orders in curved VSe_2_ monolayers can be attributed to the curvature-induced modification of the V–Se bond structure and periodic variations in V–Se bond length. A phenomenological model is established to describe the relation of the total magnetic moment in one period of a curved VSe_2_ monolayer with its curvature radius and the number of hexagonal rings that forms one period. Under a high curvature, the curved MnSe_2_ monolayer also exhibits periodic fluctuation in the magnetic moments of Mn atoms. On the contrary, no obvious periodic CDW and bond structure deviation is observed for curved CrI_3_ monolayers.

## 2. Model and methods

**Fig. 1:**
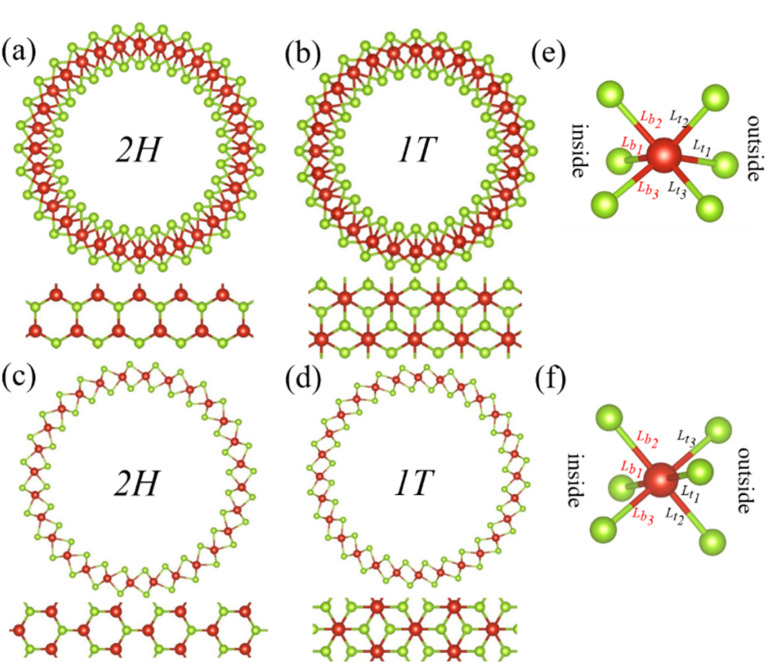
Front and top views of the relaxed structures of armchair (a) 2H-VSe_2_ and (b) 1T-VSe_2_ nanotubes, zigzag (c) 2H-VSe_2_ and (d) 1T-VSe_2_ nanotubes, and the corresponding flat monolayers. The atomic configurations of V–Se bonds of (e) 2H-VSe_2_ and (f) 1T-VSe_2_. Here *L*_t_1__, *L*_t_2__, and *L*_t_3__ denote the V–Se bonds of the outside surface of a nanotube and *L*_b_1__, *L*_b_2__, and *L*_b_3__ the V–Se bonds of the inside surface. The red and green balls are V and Se atoms, respectively.

In our model, the curved VSe_2_ monolayers were constructed by bending a flat VSe_2_ monolayer into a VSe_2_ nanotube, as shown in Fig. 1. Through this setting that was employed in the previous studies,^20,21,26,32^ a curved VSe_2_ monolayer will have a unique curvature, and the radius and curvature of a curved VSe_2_ (or a VSe_2_ nanotube) can be adjusted by changing the initial length of a flat VSe_2_ monolayer. The VSe_2_ monolayers^33^ with trigonal prismatic phase (2H) and octahedral phase (1T) were considered and bended in the armchair and zigzag directions, respectively. The periodic boundary condition was applied in the axial directions of the formed nanotubes. The atom numbers of monolayer VSe_2_ nanotubes considered in the study are given in Table S1 in the ESI.† All computations were based on the spin-resolved density functional theory (DFT) as implemented in Vienna *ab initio* simulation package code by using the projector-augmented wave method with the Perdew–Burke–Ernzerhof generalized gradient approximation (GGA) for the exchange-correlation potential.^34–36^ To properly describe the electronic and magnetic properties, we used the GGA + *U*_eff_ method introduced by Dudarev *et al.*^37^ with *U*_eff_ = 3.1 eV for V atoms, which has been used in previous studies.^38,39^ These systems were relaxed by using a conjugate-gradient method until the force on each atom was less than 0.01 eV Å^−1^. After structural relaxation, a cutoff energy of 500 eV and Γ-centered *k* points of 7 × 1 × 1 were adopted for the DFT calculations of the total energies and magnetic moments.

## 3. Results and discussion

**Fig. 2:**
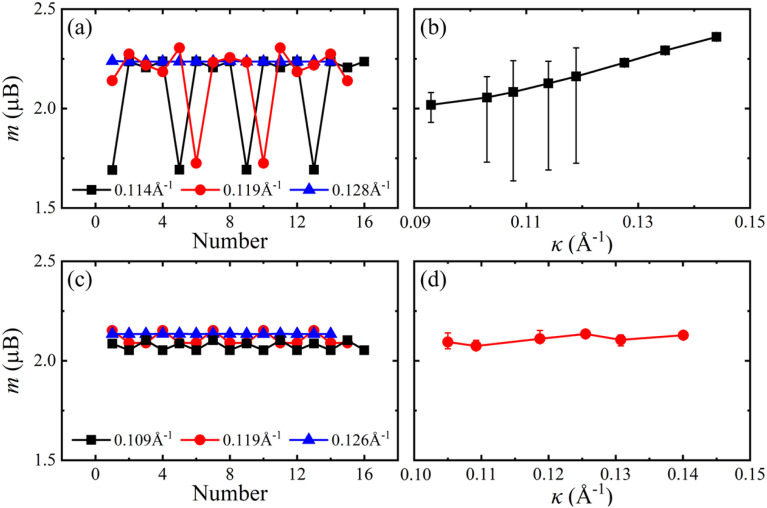
Magnetic moments *m* of V atoms along the circumference direction (left) and the average magnetic moments of all V atoms (right) for armchair ((a) and (b)) 2H-VSe_2_ and ((c) and (d)) 1T-VSe_2_ nanotubes under different curvatures. The bars in (b) and (d) denote the maximum and minimum magnetic moments of V atoms.

The ground states for flat 2H- and 1T-VSe_2_ monolayers are ferromagnetic, and the energies of antiferromagnetic (nonmagnetic) states are higher than that of ferromagnetic states. For monolayer VSe_2_ nanotubes, the ground states are still ferromagnetic and the antiferromagnetic states are not stable. The magnetic moments *m* of V atoms along the nanotube circumference direction for armchair 2H-VSe_2_ and 1T-VSe_2_ nanotubes are shown in Fig. 2(a) and (c). For a flat monolayer, the magnetic moments of V atoms are approximately uniform (see Fig. S1 in the ESI†). In contrast, the magnetic moments of V atoms of nanotubes periodically fluctuate along the circumference direction. One period of magnetic moment fluctuation in the flexural structure consists of several V–Se hexagonal rings, and the number of hexagonal rings in one period is different when a nanotube possesses a different curvature *κ* (*κ* = 1/*R*, *R* is the flexural radius). Furthermore, the average magnetic moments of all V atoms were calculated and are shown in Fig. 2. The average magnetic moments of 2H-VSe_2_ nanotubes increase with increasing curvature, and both the average magnetic moments of all V atoms and the minimum magnetic moments of V atoms are larger than the average magnetic moments of flat 2H-VSe_2_ monolayers, see Fig. S1(a).† For the 1T-VSe_2_ nanotubes, the magnetic moments of V atoms exhibit similar periodic fluctuation along the nanotube circumference direction, but the variation of average magnetic moments with curvature is not monotonic, as shown in Fig. 2(c) and (d). The average magnetic moments of all V atoms and the minimum magnetic moments of V atoms are still larger than the average magnetic moments of flat 1T-VSe_2_ monolayers, see Fig. S1(b).† In comparison with the flat states, the bending deformation enhances the magnetic moments of V atoms of armchair VSe_2_ nanotubes.

**Fig. 3:**
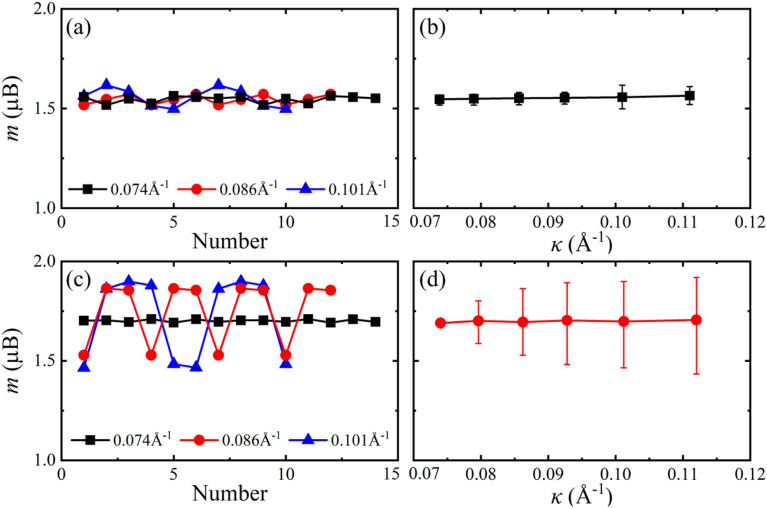
Magnetic moments *m* of V atoms along the circumference direction (left) and the average magnetic moments of all V atoms (right) for zigzag ((a) and (b)) 2H-VSe_2_ and ((c) and (d)) 1T-VSe_2_ nanotubes under different curvatures. The bars in (b) and (d) denote the maximum and minimum magnetic moments of V atoms.

When the VSe_2_ monolayers are bended in the zigzag direction, the magnetic moments of V atoms also periodically fluctuate along the nanotube circumference direction, see Fig. 3, and the average magnetic moments slightly vary with the curvature increases. The average magnetic moments of zigzag 2H-VSe_2_ and 1T-VSe_2_ nanotubes are close to that of the corresponding flat VSe_2_ monolayers, and the magnetic moments of some V atoms are lower than that of the flat states, see Fig. S2.† Therefore, the influence of bending deformation on the average magnetic moments of armchair VSe_2_ nanotubes is stronger than that of zigzag VSe_2_ nanotubes. It can be also seen from Fig. 2(d) and 3(d) that the fluctuation amplitudes of V magnetic moments of zigzag 1T-VSe_2_ nanotubes are larger than that of armchair 1T-VSe_2_ nanotubes.

**Fig. 4:**
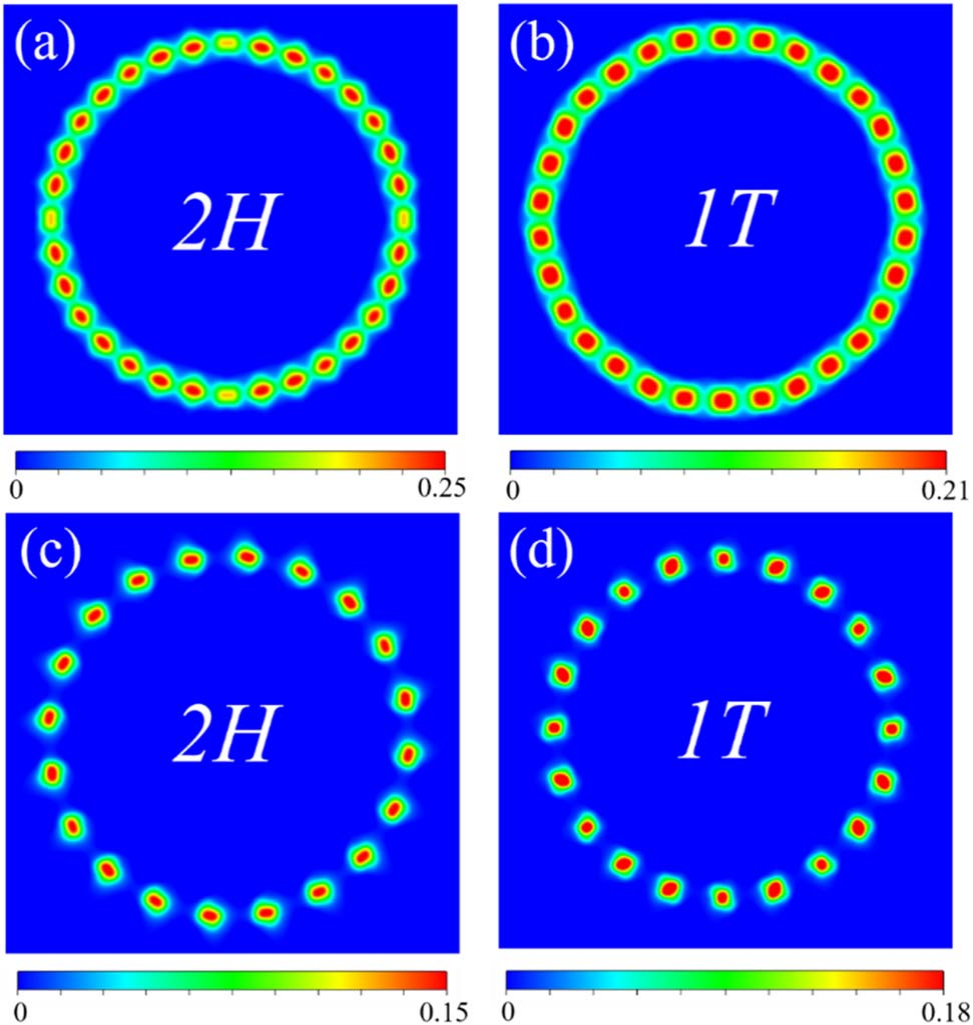
2D projection of the spin charge density differences between spin up and spin down (in units of e Å^−3^) of armchair (a) 2H-VSe_2_ nanotube with a curvature of 0.114 Å^−1^ and (b) 1T-VSe_2_ with a curvature of 0.119 Å^−1^, and zigzag (c) 2H-VSe_2_ nanotube with a curvature of 0.101 Å^−1^ and (d) 1T-VSe_2_ with a curvature of 0.101 Å^−1^.

Moreover, the CDWs form in the nanotubes according to the spin charge density differences between spin up and spin down, as shown in Fig. 4. Depending on the periodic fluctuation in the magnetic moments of V atoms, the spin charge density differences periodically vary along the circumference direction. The stronger magnetic moment fluctuation gives rise to the more obvious CDW formed in the nanotubes. For armchair nanotubes, the formation of CDWs in 2H-VSe_2_ is more favorable than that in 1T-VSe_2_. On the contrary, the formation of CDWs in 1T-VSe_2_ becomes more favorable for zigzag nanotubes. Previous works^16^ have revealed the existence of CDWs in flat VSe_2_ monolayers and found that the presence of CDWs induced by structural distortion suppresses the intrinsic ferromagnetism of VSe_2_. Our DFT results further demonstrate that the CDWs can be caused in VSe_2_ monolayers through bending deformation.

**Fig. 5:**
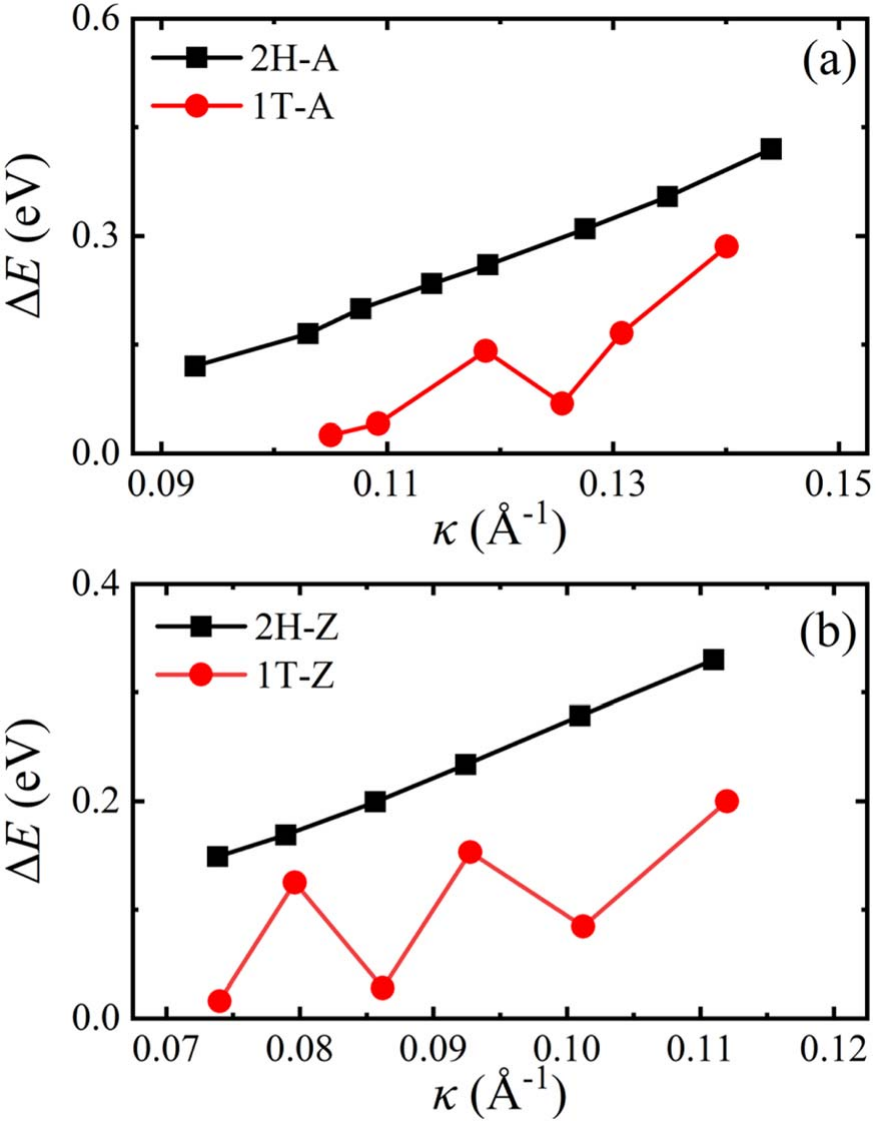
Bending energies Δ*E*(*κ*) of armchair (a) and zigzag (b) VSe_2_ nanotubes with curvature.

To better understand the effect of bending deformation on the bond structure of VSe_2_ monolayers, the bending energies Δ*E*(*κ*) for 2H-VSe_2_ and 1T-VSe_2_ nanotubes were calculated by 
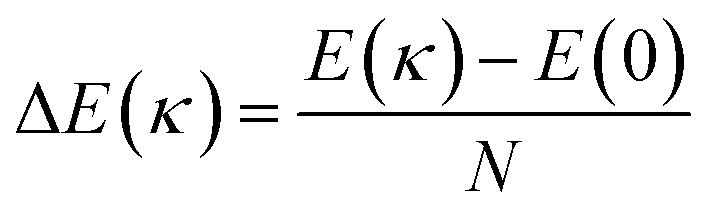
, where *E*(*κ*) is the total energy of a nanotube, *E*(0) is the total energy of the corresponding flat monolayer obtained from the nanotube, and *N* is the total number of V atoms. As shown in Fig. 5, the bending energies of armchair and zigzag 2H-VSe_2_ nanotubes monotonically increase with the curvature increases. This is consistent with the prediction of elastic mechanics for a bended thin film. For the 1T-VSe_2_ nanotubes, the bending energies exhibit nonmonotonic variations with curvature no matter whether the monolayers are bended in the armchair or zigzag direction. The significant difference in bending energies between 2H-VSe_2_ and 1T-VSe_2_ nanotubes indicates that the response of V–Se bond structure and atomic configuration to bending deformation for 2H-VSe_2_ and 1T-VSe_2_ will be different.

**Fig. 6:**
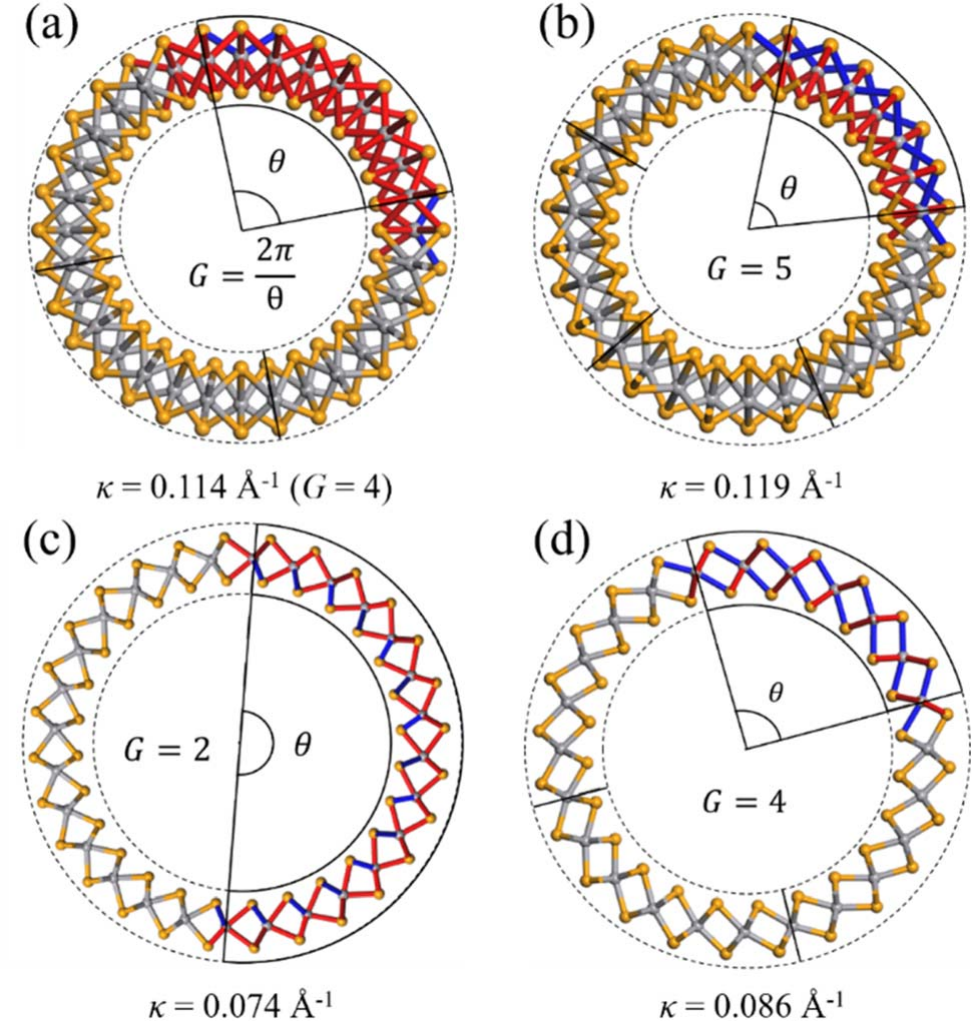
Periodic bond structures of armchair (a) 2H-VSe_2_ and (b) 1T-VSe_2_ nanotubes, and zigzag (c) 2H-VSe_2_ and (d) 1T-VSe_2_ nanotubes. Here, the red and blue bonds represent the elongation and compression of V–Se bonds with respect to the V–Se bonds in the flat state, respectively.

**Fig. 7:**
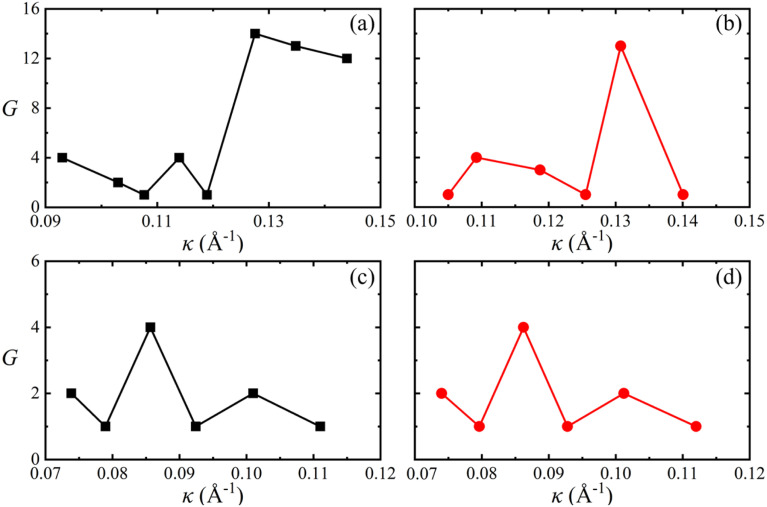
Periodic numbers *G* for armchair (a) 2H-VSe_2_ and (b) 1T-VSe_2_ nanotubes, and zigzag (c) 2H-VSe_2_ and (d) 1T-VSe_2_ nanotubes with curvature.

One V atom bonds with six Se atoms, and there are three V–Se bonds outside and three V–Se bonds inside the curved monolayer, as shown in Fig. 1. The length of V–Se bonds is altered by bending deformation. The elongation and compression of V–Se bonds in the nanotubes also exhibit periodic fluctuation along the circumference direction, as shown in Fig. 6. The period number *G* is defined by *G* = 2π/*θ*, where *θ* is the angle corresponding to one period. It should be mentioned that the period number *G* for bond structure is the same as that of the magnetic moments of V atoms. As shown in Fig. 7, the variations of period numbers *G* for both armchair and zigzag VSe_2_ nanotubes are not monotonic as the curvature increases.

**Fig. 8:**
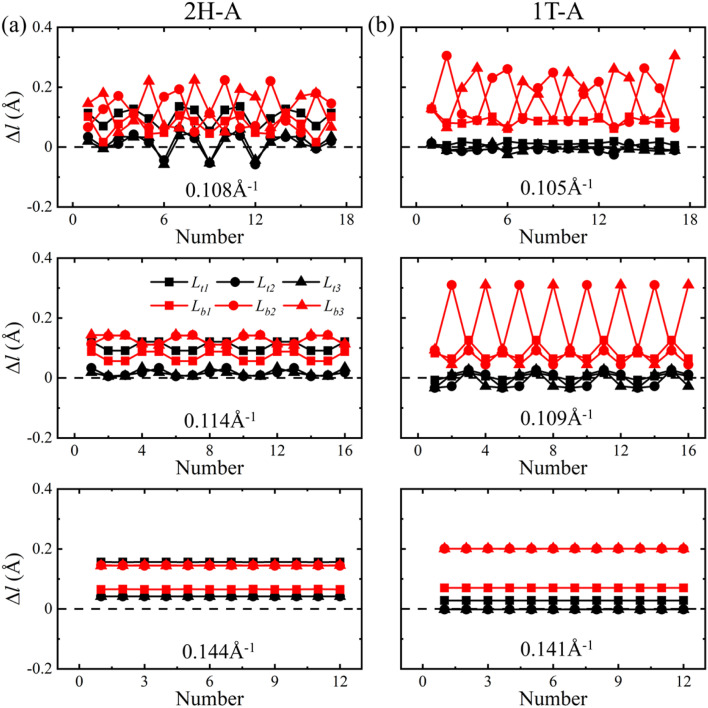
Deviations of V–Se bond lengths Δ*l* along the circumference direction for armchair 2H-VSe_2_ (left) and 1T-VSe_2_ (right) nanotubes under different curvatures.

**Fig. 9:**
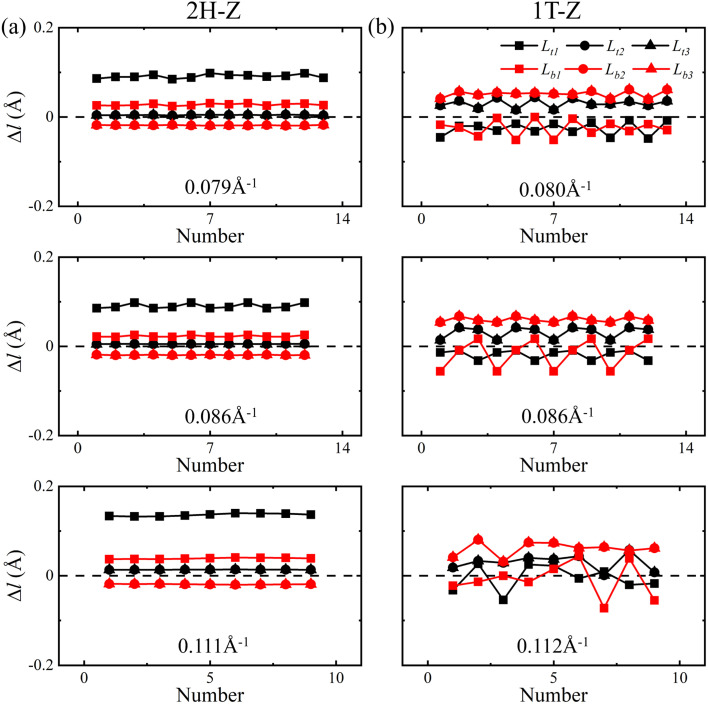
Deviations of V–Se bond lengths Δ*l* along the circumference direction for zigzag 2H-VSe_2_ (left) and 1T-VSe_2_ (right) nanotubes under different curvatures.

In order to further elucidate the change in V–Se bonds under bending deformation, the bond length deviations Δ*l* with respect to the bond length (2.53 Å) of a unit cell in the flat state were calculated. Here the positive values of Δ*l* represent the bond elongation and the negative values represent the bond compression. As shown in Fig. 8 and 9, the bond length deviations exhibit periodic fluctuation along the circumference direction, and the periods are the same as that of magnetic moments of V atoms. When bended in the armchair direction, the inside V–Se bonds of 2H–VSe_2_ and 1T-VSe_2_ nanotubes are all stretched, while some outside V–Se bonds are stretched and some are compressed, see Fig. 8. The magnetic moment of a V atom is usually determined by the competition of two distinct interactions between the V and Se atoms: the covalent bonding interaction and the ionic bonding interaction.^40^ The elongating of a V–Se bond reduces the covalent bonding interaction and increases the ionic bonding interaction. Meanwhile, the relative enhancement of the ionic bond interaction between the V and Se atoms increases the distribution of unpaired electrons on the V atom, which accordingly increases the magnetic moment of the V atom. The compression of a V–Se bond gives rise to an opposite contribution to the ionic bond interaction and the magnetic moment of the V atom. The length deviations in the six V–Se bonds connecting one V atom are different under bending deformation. For armchair 2H-VSe_2_ and 1T-VSe_2_ nanotubes, most V–Se bonds are stretched (see Fig. 8), which accordingly enhances the magnetism of V atoms. This mainly contributes to the increase of magnetic moments of the V atom shown in Fig. 2.

On the other hand, some of the inside and outside V–Se bonds are stretched and some are compressed when the VSe_2_ monolayers are bended in the zigzag direction, as shown in Fig. 9. The competition between bond elongation and compression leads to the magnetic moment enhancement of some V atoms and reduction of some V atoms for zigzag nanotubes. It can be concluded from these results that the periodic fluctuation in the magnetic moments of V atoms stems from the periodic fluctuation of V–Se bond length deviations. No matter 2H-VSe_2_ or 1T-VSe_2_ nanotubes, a larger fluctuation of bond length deviations will lead to a higher fluctuation of magnetic moments.

**Fig. 10:**
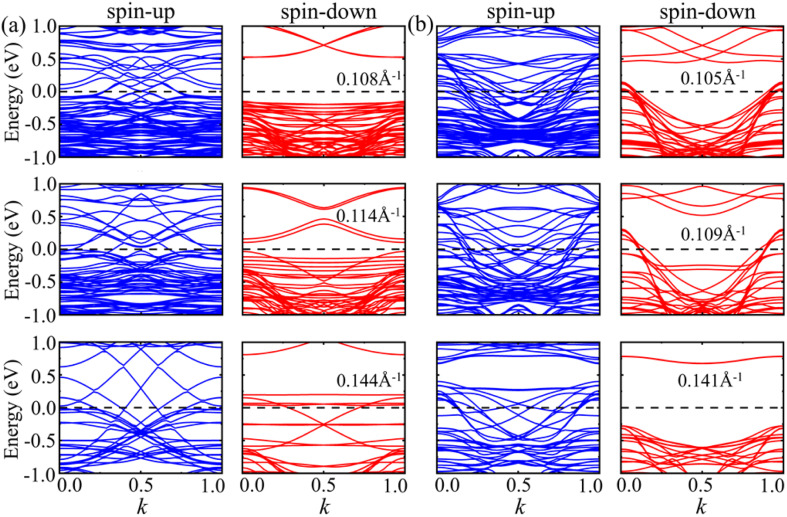
Energy bands of spin-up and spin-down for armchair (a) 2H-VSe_2_ and (b) 1T-VSe_2_ nanotubes under different curvatures. The Fermi level is set to zero.

**Fig. 11:**
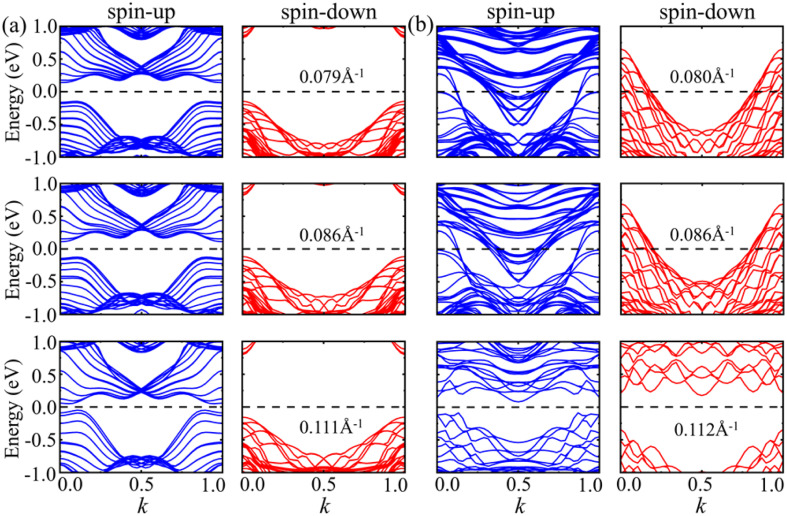
Energy bands of spin-up and spin-down for zigzag (a) 2H-VSe_2_ and (b) 1T-VSe_2_ nanotubes under different curvatures. The Fermi level is set to zero.

For armchair 2H- and 1T-VSe_2_ nanotubes, it can be seen from the band structures shown in Fig. 10 that the spin-up of armchair 2H- and 1T-VSe_2_ nanotubes is metallic. The band gap of spin-down of 2H-VSe_2_ decreases with the increase of curvature and the spin-down gradually transforms from semiconducting into metallic. On the contrary, the spin-down of 1T-VSe_2_ transforms from metallic into semiconducting with the increase of curvature, exhibiting a sudden change. For zigzag 2H-VSe_2_ nanotubes, the band gap of spin-up decreases with the increase of curvature but the spin-down remains semiconducting, as shown by Fig. 11(a). In contrast, both the spin-up and spin-down of zigzag 1T-VSe_2_ exhibit a sudden change from metallic into semiconducting under a large curvature, as shown by Fig. 11(b). The sudden change in the energy band structures of armchair and zigzag 1T-VSe_2_ nanotubes is consistent with the nonmonotonic variations of bending energies shown in Fig. 5. It can be also seen from Fig. S3† that the spin-resolved local density of states (LDOS) of the V atoms in one period for the armchair and zigzag VSe_2_ nanotubes are different, which is consistent with the curvature induced deviations in the bond structure and magnetic moment of V atom.

To give a more suitable description of the relationship between the magnetism and curvature for curved VSe_2_ monolayers, we define two parameters: the total magnetic moment *m*_t_ in one period, and the number of hexagonal rings *n* that exist in one period. As shown by Fig. S4 in the ESI,† the total magnetic moments *m*_t_ of armchair 2H-VSe_2_ nanotubes can be well fitted by *m*_t_ = *AR* + *Bn* + *C*, where *A*, *B* and *C* are fitting parameters. The unit of the parameter *A* is μB nm^−1^. *A* is related to the nanotube radius *R*, which reflects the influence of curvature on the total magnetic moment in one period. A large value of *A* means a stronger influence from curvature. The parameter *B* with the unit of μB reflects the influence of bond structure change induced by bending deformation on the magnetism. The unit of the parameter *C* is μB, and *C* is constant under different curvatures. For other VSe_2_ nanotubes, their total magnetic moment *m*_t_ in one period can also be fitted by the equation *m*_t_ = *AR* + *Bn* + *C*, see Table S2 in the ESI.† These results further demonstrate that the magnetism of curved VSe_2_ monolayers is not only determined by the curvature but also related to the V–Se bond structure in one period.

Moreover, the influence of bending deformation on the magnetic properties and bond structure of MnSe_2_ and CrI_3_ monolayers has been investigated by using the same procedure. For most curved 1T-MnSe_2_ monolayers, the magnetic moments of Mn atoms are slightly changed by the bending deformation and no CDW is observed, as shown by Fig. S6 in the ESI.† However, the bending deformation still can induce obvious fluctuation in the magnetic moments of Mn atoms when the curvature is large enough and the monolayer is bended in the armchair direction. The atomic configuration and Mn–Se bond structure of a 1T-MnSe_2_ monolayer are similar to that of a 1T-VSe_2_ monolayer. The fluctuation in the magnetic moments of Mn atoms is mainly attributed to the fluctuation in the length deviations of Mn–Se bonds under bending deformation, see Fig. S8.† For curved CrI_3_ monolayers, the bending deformation increases the magnetic moments of Cr atoms in comparison with the flat states, but no fluctuation in the magnetic moments of Cr atoms and length deviations of Cr–I bonds is observed, as shown by Fig. S10 and S12 in the ESI.† The CDW is also not observed for curved CrI_3_ monolayers. The atomic configuration and Cr–I bond structure of a CrI_3_ monolayer are completely different from that of a VSe_2_ monolayer, which leads to the different response of magnetic properties to bending deformation.

## 4. Conclusions

In summary, our extensive DFT calculations reveal the significant periodic fluctuation in the magnetic moments of V atoms for curved VSe_2_ monolayers, and the periodic CDWs are also observed. The periodic fluctuation in the magnetic moments of V atoms mainly stems from the periodic fluctuation of V–Se bond length deviations. The bending deformation enhances the magnetic moments of V atoms of armchair VSe_2_ nanotubes as most V–Se bonds are elongated. A phenomenological model is established to describe the relation of the magnetism of a curved VSe_2_ monolayer with its curvature radius and bond structure. The curved MnSe_2_ monolayer exhibits periodic fluctuation in the magnetic moments of Mn atoms when it is bended under a high curvature. No obvious periodic CDW and bond structure deviation is observed for curved CrI_3_ monolayers. Our results unveil the different response of magnetic 2D monolayers to bending deformation and provide a possible way to develop functional magnetic devices by mechanical design.

## Conflicts of interest

There are no conflicts to declare.

## Supplementary Material

RA-013-D3RA01319G-s001
