# Supplementary material for: Magnetism in curved VSe2 monolayers
Source: RSC Adv. 2023 Mar 14;13(12):8307–16. doi: 10.1039/d3ra01319g (PMC10012335; doi:10.1039/d3ra01319g)
Supplement: RA-013-D3RA01319G-s001 [file RA-013-D3RA01319G-s001.pdf]

Electronic Supplementary Material (ESI) for RSC Advances.
This journal is © The Royal Society of Chemistry 2023

Supporting Information for Magnetism in curved VSe_2 monolayers

Kexin Mi, Yufeng Guo*

State Key Laboratory of Mechanics and Control of Mechanical Structures and MOE Key Laboratory for Intelligent Nano Materials and Devices, College of Aerospace Engineering, Nanjing University of Aeronautics and Astronautics, Nanjing, 210016, China

* *yfguo@nuaa.edu.cn*

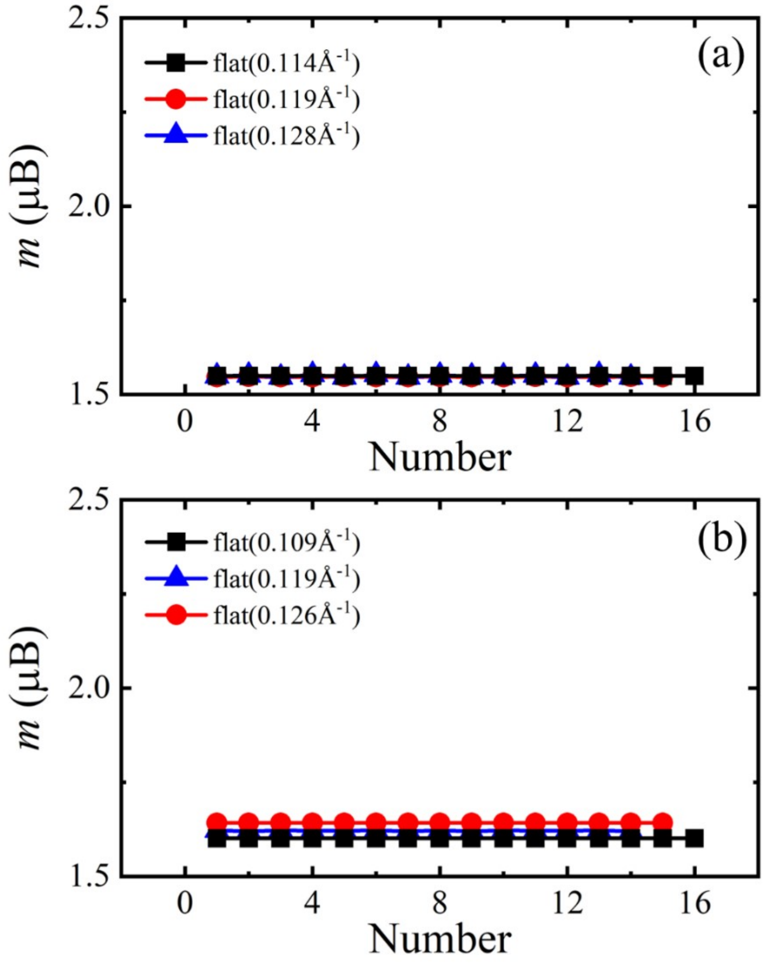

Figure S1. Magnetic moments *m* of V atoms along the circumference direction for the corresponding flat states of armchair (a) 2H-VSe_2 and (b) 1T-VSe_2 nanotubes with different curvatures.

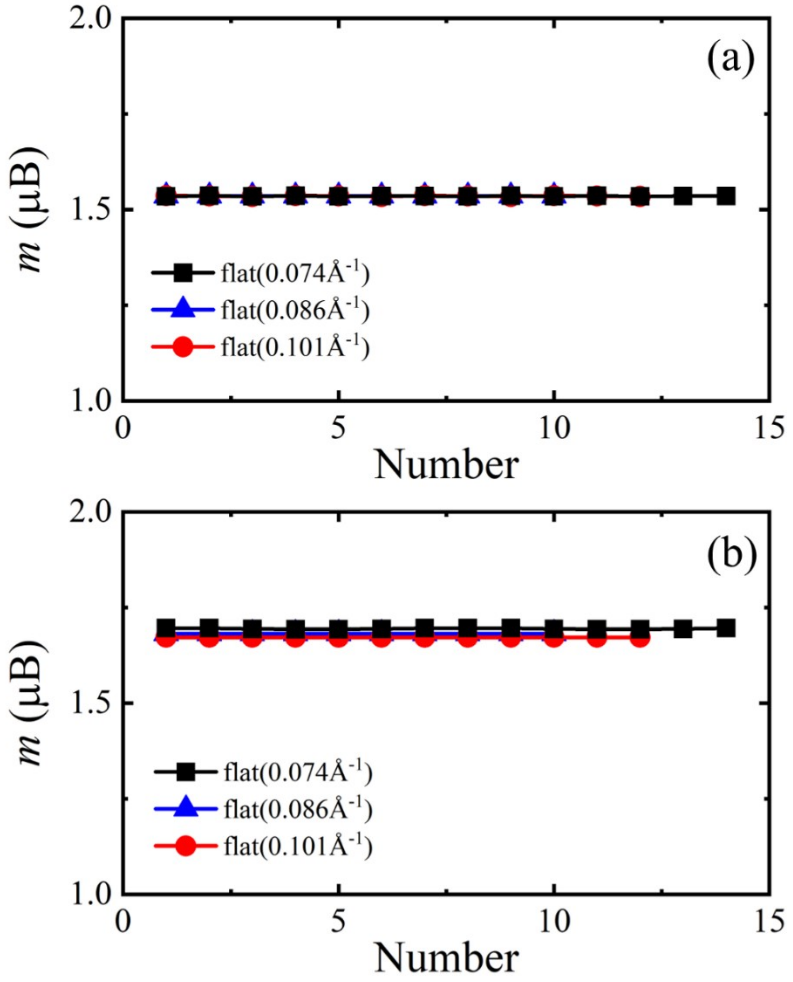

Figure S2. Magnetic moments *m* of V atoms along the circumference direction for the corresponding flat states of zigzag (a) 2H-VSe_2 and (b) 1T-VSe_2 nanotubes with different curvatures.

Table S1. The atom numbers of VSe_2 nanotubes.

		Armchair							
2H	κ(Å^{-1})	0.093	0.103	0.108	0.114	0.119	0.128	0.135	0.144
	N(V/Se)	40/80	36/72	34/68	32/64	30/60	28/56	26/52	24/48
1T	κ(Å^{-1})	0.105	0.109	0.119	0.126	0.131	0.141		
	N(V/Se)	34/68	32/64	30/60	28/56	26/52	24/48		
		Zigzag							
2H	κ(Å^{-1})	0.074	0.079	0.086	0.092	0.101	0.111		
	N(V/Se)	28/56	26/52	24/48	22/44	20/40	18/36		
1T	κ(Å^{-1})	0.074	0.080	0.086	0.093	0.101	0.112		
	N(V/Se)	28/56	26/52	24/48	22/44	20/40	18/36		

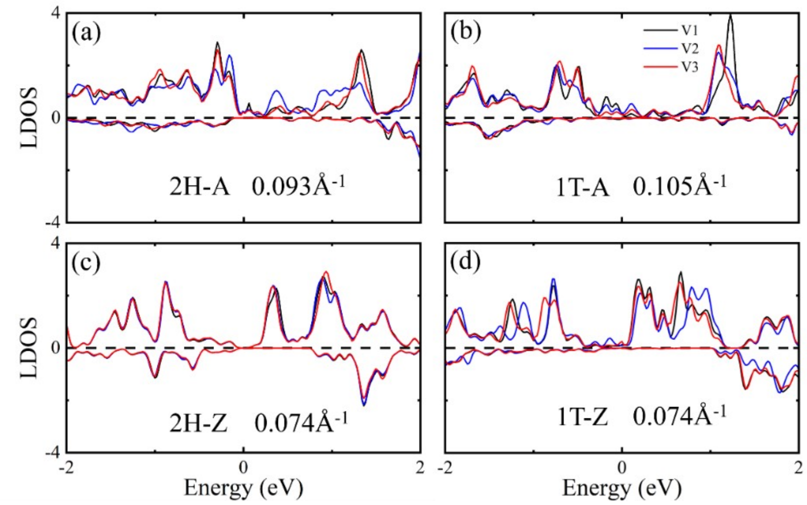

Figure S3. The spin-resolved LDOS of *d* orbitals of the V atoms in one period for different VSe_2 nanotubes.

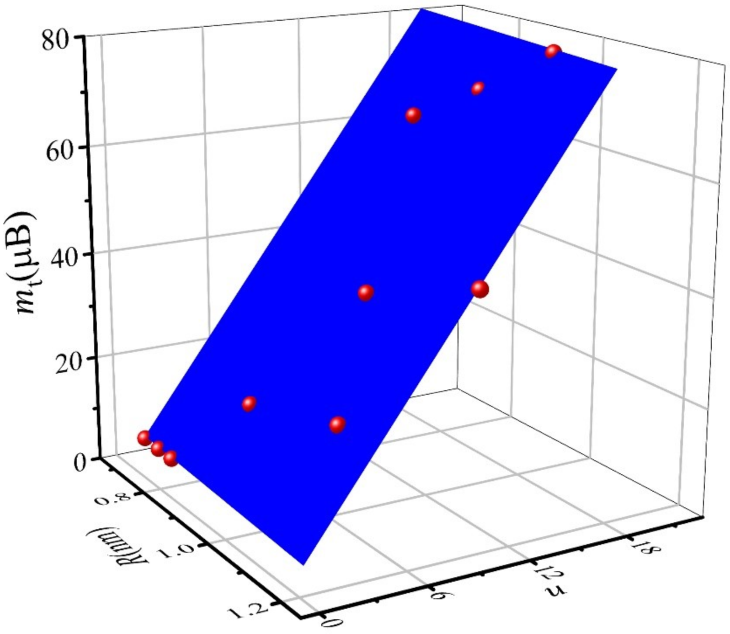

Figure S4. Total magnetic moments m_t of one period of armchair 2H-VSe_2 nanotubes with different curvature radii and hexagonal ring number *n*. The blue plane is the fitting of total magnetic moments.

Figure S3 shows the total magnetic moment m_t in one period for armchair 2H-VSe_2 nanotubes. The data can be approximately fitted by a function $m_t = AR + Bn + C$, where

A = -4.79 μB/nm, B = 4.22 μB, and C = 4.24 μB. Additionally, the relationship between magnetism and curvature for other phases and bending directions have the same formula and the only difference is the value of the parameters A, B, and C.

Table S2. The value of the parameters A, B, and C.

		A(μB/nm)	B(μB)	C(μB)
Armchair	2H	-4.79	4.22	4.24
	1T	-0.44	3.1	0.53
Zigzag	2H	-1.53	4.2	1.29
	1T	-0.32	3.4	0.39

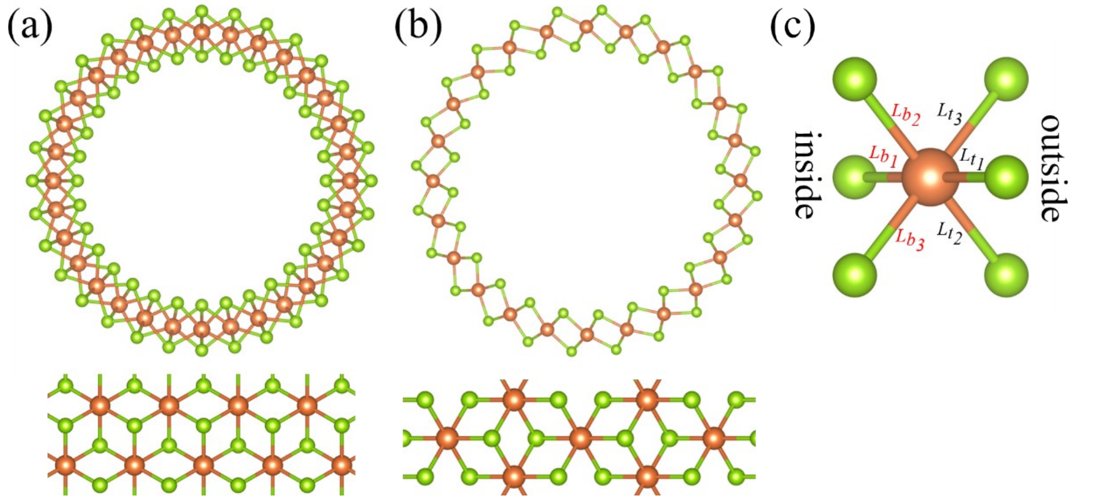

Figure S5. Front and top views of the relaxed structures of (a) armchair and (b) zigzag 1T-$MnSe_2$ nanotubes and the corresponding flat monolayers. (c) The atomic configurations of Mn-Se bonds of 1T-$MnSe_2$. Here L_{t1}, L_{t2}, and L_{t3} denote the Mn-Se bonds of the outside surface of a nanotube, and L_{b1}, L_{b2}, and L_{b3} are the Mn-Se bonds of the inside surface. The orange and green balls are Mn and Se atoms, respectively.

To properly describe the electronic and magnetic properties, we used the GGA+U_{eff} method introduced by Dudarev et al.[1] with U_{eff} = 3.8 eV for Mn atoms, which has been used in previous studies[2, 3]. These systems were relaxed by using a conjugate-gradient method until the force on each atom was less than 0.01 eV/Å. After structural relaxation, a cutoff energy of 500 eV and Γ-centered kpoints of 7 × 1 × 1 were adopted for the DFT calculations of the total energies and magnetic moments.

Table S3. The atom numbers of 1T-$MnSe_2$ nanotubes.

	Armchair					Zigzag				
κ(Å^{-1})	0.104	0.112	0.123	0.131	0.143	0.082	0.089	0.097	0.107	0.119
N(Mn/Se)	24/48	22/44	20/40	18/36	16/32	32/64	30/60	28/56	26/52	24/48

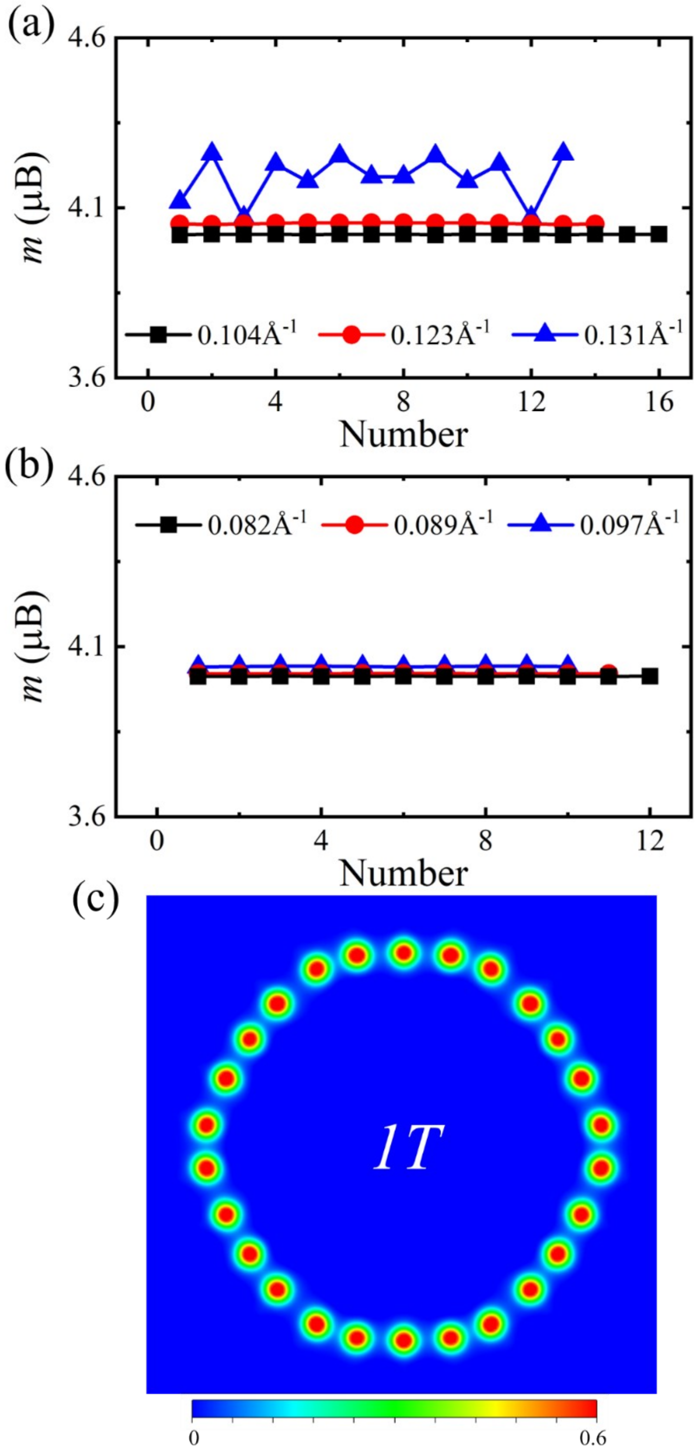

Figure S6. Magnetic moments *m* of Mn atoms along the circumference direction for (a) armchair and (b) zigzag 1T-$MnSe_2$ nanotubes under different curvatures. (c) 2D projection of the spin charge density differences between spin up and spin down (in units of e/Å^3) of an armchair 1T-$MnSe_2$ nanotube with a curvature of 0.131 Å^{-1}.

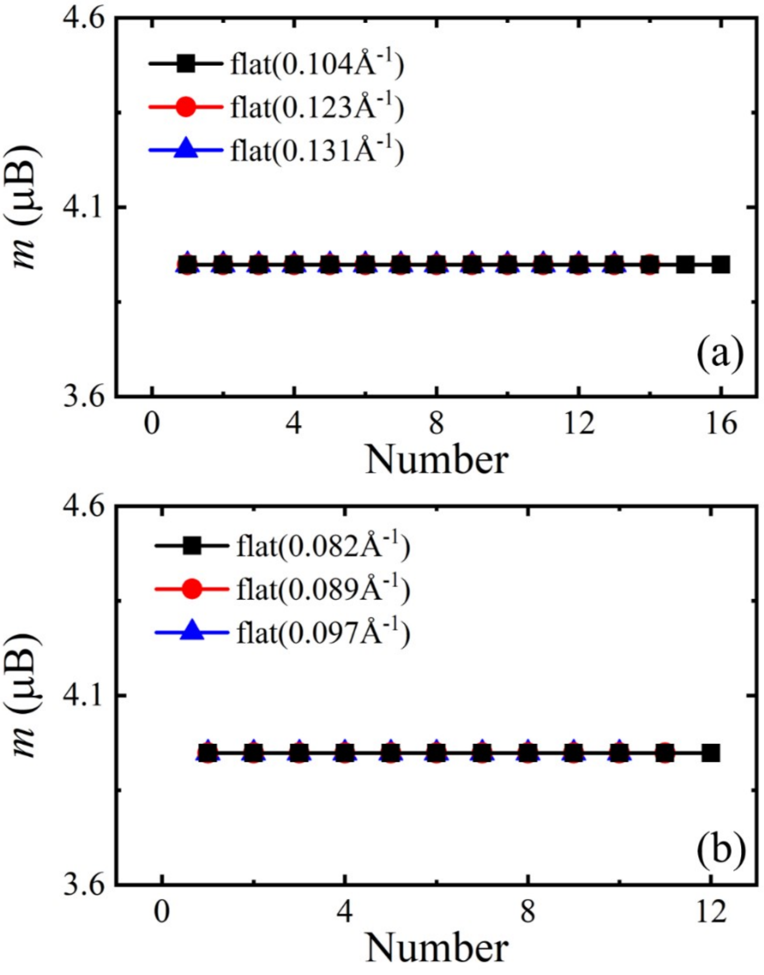

Figure S7. Magnetic moments *m* of Mn atoms along the circumference direction for the corresponding flat of (a) armchair and (b) zigzag 1T-$MnSe_2$ nanotubes.

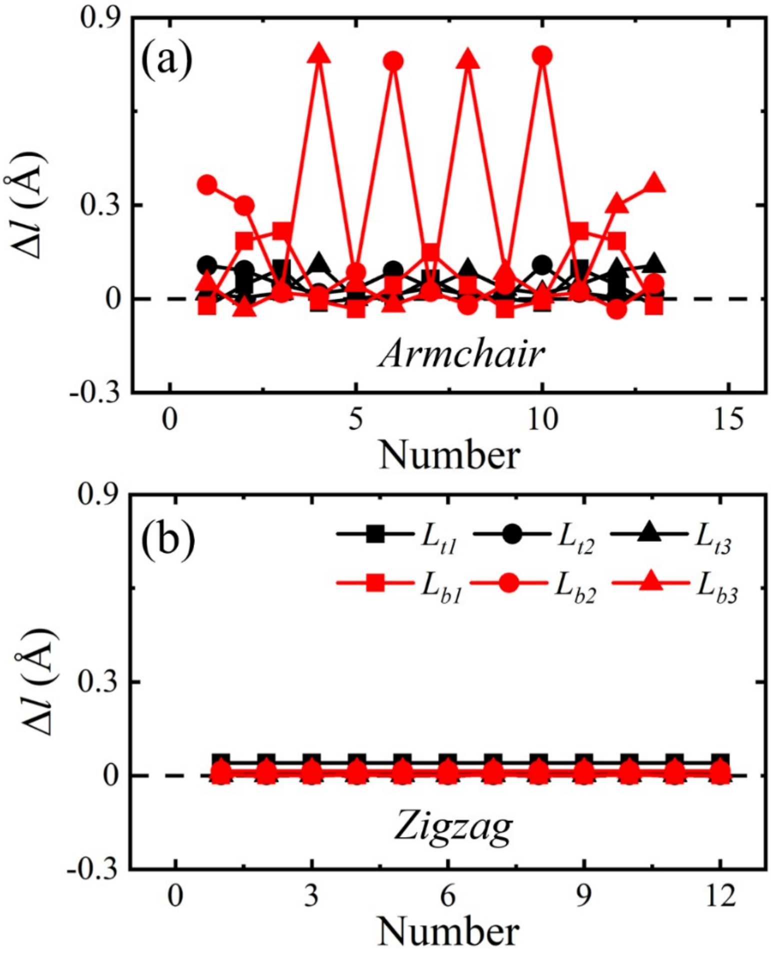

Figure S8. Deviations of Mn-Se bond lengths Δ*l* along the circumference direction for (a) armchair (0.131$Å^{-1}$) and (b) zigzag (0.082$Å^{-1}$) 1T-$MnSe_2$ nanotubes.

The bond length deviations Δ*l* with respect to the bond length (2.55Å) of a unit cell in the flat state were calculated. Here the positive values of Δ*l* represent the bond elongation and the negative values represent the bond compression.

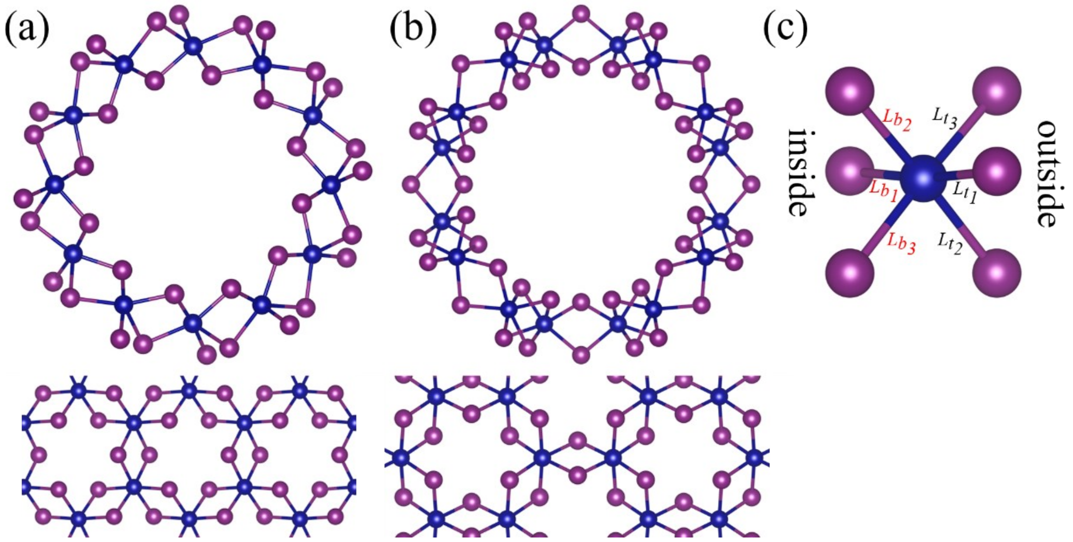

Figure S9. Front and top views of the relaxed structures of (a) armchair and (b) zigzag CrI_3 nanotubes and the corresponding flat monolayers. (c) The atomic configurations of Cr-I bonds of CrI_3. Here L_{t1}, L_{t2}, and L_{t3} denote the Cr-I bonds of the outside surface of a nanotube, and L_{b1}, L_{b2}, and L_{b3} are the Cr-I bonds of the inside surface. The blue and purple balls are Cr and I atoms, respectively.

To properly describe the electronic and magnetic properties, we used the GGA+U_{eff} method introduced by Dudarev et al.[1] with U_{eff} = 3.5 eV for Cr atoms, which has been used in previous studies[2, 3]. These systems were relaxed by using a conjugate-gradient method until the force on each atom was less than 0.01 eV/Å. After structural relaxation, a cutoff energy of 500 eV and Γ-centered kpoints of 3 × 1 × 1 were adopted for the DFT calculations of the total energies and magnetic moments.

Table S4. The atom numbers of CrI_3 nanotubes.

	Armchair			Zigzag		
κ(Å^{-1})	0.105	0.118	0.135	0.071	0.082	0.118
N(Cr/I)	32/96	28/84	24/72	21/63	18/54	12/36

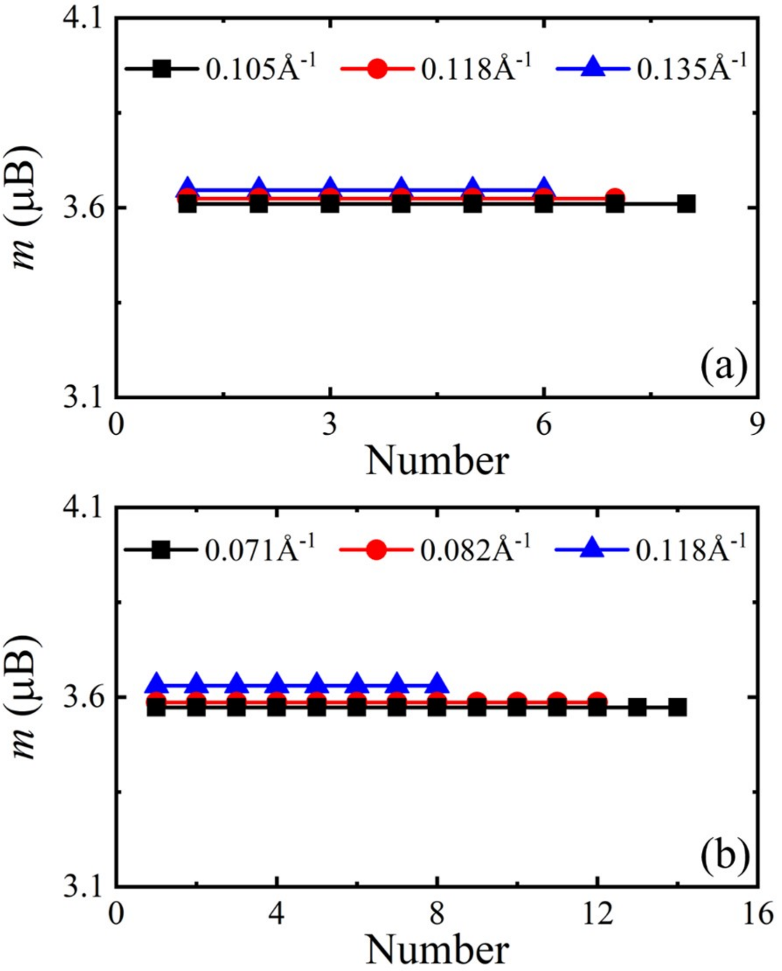

Figure S10. Magnetic moments *m* of Cr atoms along the circumference direction for (a) armchair and (b) zigzag CrI_3 nanotubes under different curvatures.

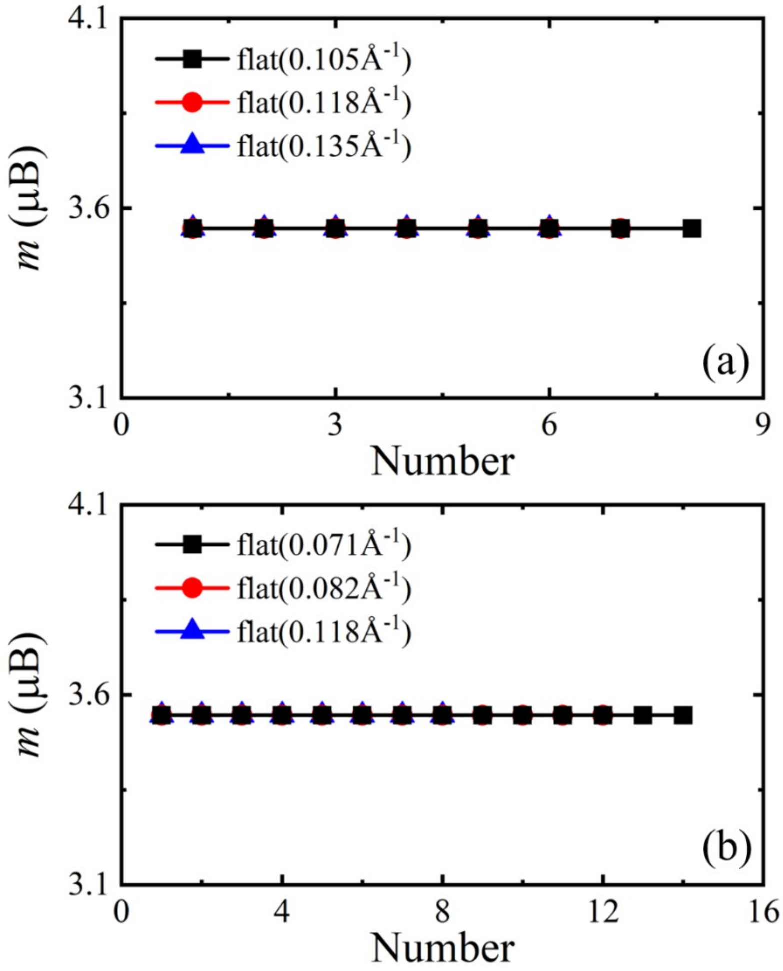

Figure S11. Magnetic moments *m* of Cr atoms along the circumference direction for the corresponding flat states of (a) armchair and (b) zigzag CrI_3 nanotubes.

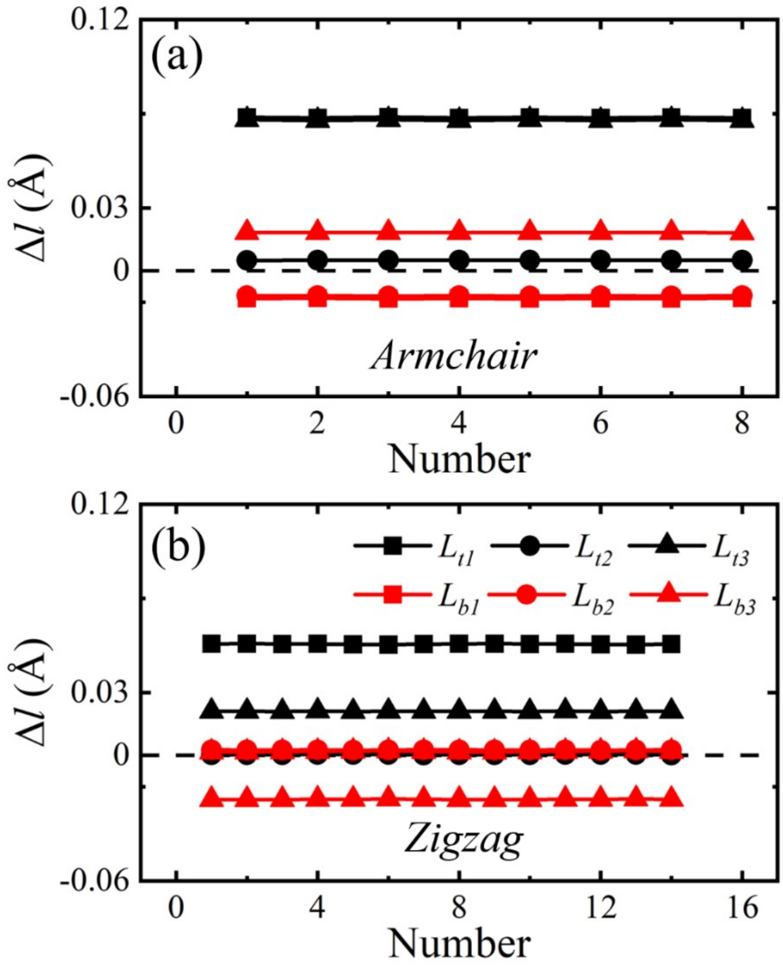

Figure S12. Deviations of Cr-I bond lengths Δ*l* along the circumference direction for (a) armchair (0.135Å^{-1}) and (b) zigzag (0.071Å^{-1}) CrI_3 nanotubes.

The bond length deviations Δ*l* with respect to the bond length (2.78Å) of a unit cell in the flat state were calculated. Here the positive values of Δ*l* represent the bond elongation and the negative values represent the bond compression.

References

1. S. L. Dudarev, G. A. Botton, S. Y. Savrasov, C. Humphreys and A. P. Sutton, *Phys. Rev. B*, 1998, **57**, 1505.
2. A. Jain, G. Hautier, C. J. Moore, S. Ping Ong, C. C. Fischer, T. Mueller, K. A. Persson and G. Ceder, *Comput. Mater. Sci.*, 2011, **50**, 2295-2310.
3. L. Wang, T. Maxisch and G. Ceder, *Phys. Rev. B*, 2006, **73**, 195107.
